# Atezolizumab-induced encephalitis in a patient with metastatic breast cancer: a case report and review of neurological adverse events associated with checkpoint inhibitors

**DOI:** 10.4322/acr.2021.261

**Published:** 2021-04-19

**Authors:** Rita Nader, Esther Tannoury, Tamina Rizk, Hady Ghanem

**Affiliations:** 1 Lebanese American University Medical Center – Rizk Hospital, Beirut, Lebanon

**Keywords:** Atezolizumab, Immunotherapy, Encephalitis, Breast Neoplasms, Neurology

## Abstract

Immune-mediated encephalitis as an adverse event due to checkpoint inhibitors is very rare. We describe herein the case of a 38-year-old woman with metastatic triple-negative breast cancer who developed seizures and somnolence twelve days after receiving the first dose of Atezolizumab. Work up ruled out all infectious etiologies, and the patient was eventually diagnosed with immune-mediated meningoencephalitis. Symptoms recovered with a high-dose of steroids, and she was found to have an excellent response on follow-up imaging, which raised the question of whether a relationship exists between the occurrence, and severity of the adverse event and the response to treatment. Only a few other cases of atezolizumab–related encephalitis have been published. Early recognition and treatment are crucial; the reason why we are describing this case along with a review of the literature and a review on all the neurological immune-related adverse events due to the different checkpoint inhibitors.

## INTRODUCTION

In the past decade, immune checkpoint inhibitors (ICI) have changed the treatment paradigm of the solid tumors. The first breakthrough in cancer immunotherapy was the discovery of the cytotoxic T-lymphocyte-associated protein 4 (CTLA-4), and the first antibody directed against the CTLA-4, Ipilimumab, was approved in 2011 for the treatment of advanced Melanoma.^1^ Other checkpoint inhibitors targeting the interaction between the programmed cell death-1 (PD-1) on T-cell receptors and its ligand, the programmed death-ligand 1 (PDL-1) on tumor cells were then studied. These include (but are not limited to) Nivolumab and Pembrolizumab, which are PD-1 blockers, and Atezolizumab that blocks the PDL-1. By blocking these proteins, the inhibitory pathway is inactivated, thus unleashing the T-cells against antigens of the neoplastic cells. However, this over-exaggerated activation of the immune system can come with a price, and many immune-related adverse events (irAE) have been observed in clinical trials and in clinical practice. Neurologic irAE occurs less frequently than other irAE; however, it can, at times, be severe and sometimes fatal. We report a case of meningoencephalitis as an adverse event from Atezolizumab during the treatment of advanced metastatic triple-negative breast cancer along with a review of possible neurological irAEs emerging with the growing use of ICI and immunotherapy in general.

## CASE REPORT

A 38-year-old woman was diagnosed with locally advanced triple-negative breast cancer. She had no previous medical problems, no family history of malignancy, and was *BRCA* negative despite her young age. She was initially treated by a subcutaneous mastectomy with axillary lymph node dissection and was found to have a pT3N1 disease. She had no metastatic disease and thus was staged as IIIA and received adjuvant chemotherapy with Adriamycin and Cyclophosphamide followed by Docetaxel, followed by adjuvant radiotherapy.

Two years later, she was found to have recurrent metastatic disease, mainly in the right upper lung lobe and hilum, confirmed to be triple-negative breast adenocarcinoma by fine-needle aspiration (FNA) by ultrasound (EBUS). Since then, she received several lines of treatment with initially good response followed by a progression of the disease. These included Carboplatin and Paclitaxel, Bevacizumab, and Capecitabine, then Eribulin. She eventually progressed on Eribulin, with increasing cough and dyspnea, and a new lesion in the right lung compressing the airways.

PDL-1 testing on the FNA at that time was negative. However, the patient had an excellent performance status, and in view of very limited options, the decision was made to start with stereotactic radiation for symptomatic relief along with Atezolizumab. Nab-Paclitaxel was planned to be added to Atezolizumab once radiotherapy was completed. This was based on a recent study that showed a progression-free survival benefit with the addition of Atezolizumab to Nab-Paclitaxel in Advanced Triple-Negative Breast Cancer compared to Nab-paclitaxel alone. Patients included in the study who were PDL1 negative could still benefit from the addition of Atezolizumab, though to a lower extent than PDL-1 positive patients.^2^

**Figure 1:**
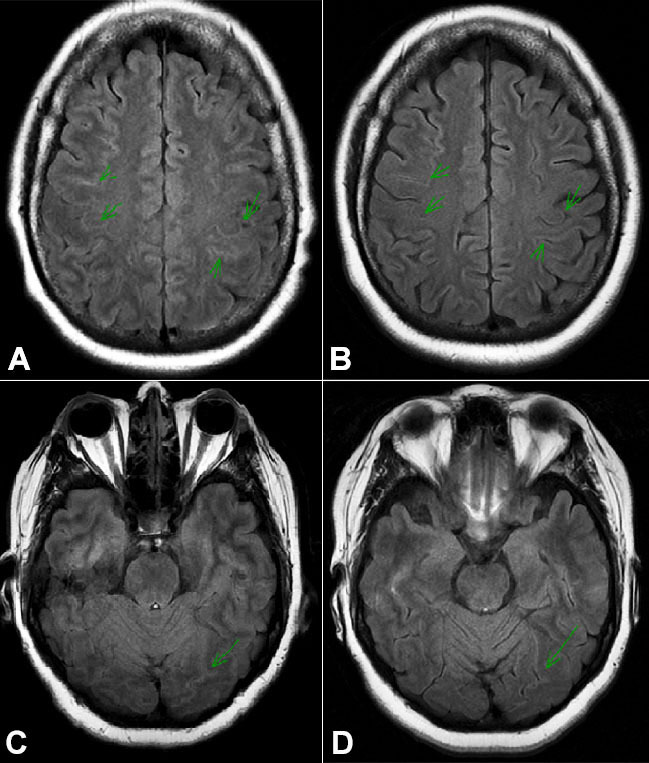
Axial T2 FLAIR MRI sequences of the brain show a diffuse subtle hyper-intense signal of the sulci when done two weeks after the patient received Atezolizumab (**A** and **C**), suggestive of the leptomeningeal irritation (could be related to leptomeningeal spread of disease, meningitis, or an inflammatory process), and interval resolution of the sulcal hyper-intense signal after a few months (**B** and **D**). There was no abnormal intra-parenchymal signal on FLAIR.

Ten days after receiving the first dose of Atezolizumab, she developed fever refractory to antibiotics reaching 40 degrees Celsius. The fever was associated with nausea, vomiting, and headaches. Laboratory workup showed only leukopenia. Our working diagnosis was infectious versus immune-mediated adverse effects from the immunotherapy. She was started on broad-spectrum antibiotics and antivirals. Workup at that time had shown negative IgM serologies for Parvovirus, CMV, and EBV, negative influenza rapid test, negative urine, and blood cultures, a low TSH but normal free T4 and T3, and a normal morning cortisol level. CT scan of the chest did not show any infiltrates or signs of infection. A couple of days later, the patient’s mental status acutely deteriorated; she became more somnolent and disoriented and then developed tonic-clonic seizures. She got intubated for airway protection and transferred to the intensive care unit (ICU). A magnetic resonance imaging (MRI) of the brain showed moderate diffuse leptomeningeal enhancement bilaterally (Figure 1). A lumbar puncture (LP) showed 15 white blood cells/mm^3^ with lymphocytosis of 80% lymphocytes, of which 26% were atypical, 79 mg/dl glucose, and 60 mg/dl proteins. Cerebrospinal fluid (CSF) culture was negative, and cytology showed inflammatory cells only with no malignant cells. Polymerase chain reaction (PCR) multiplex for viral infections on CSF (adenovirus, CMV, EBV, HSV1, HSV2, Varicella-Zoster Virus, Enterovirus, Parechovirus, HHV6, HHV7, and parvovirus B19) was also negative.

**Figure 2:**
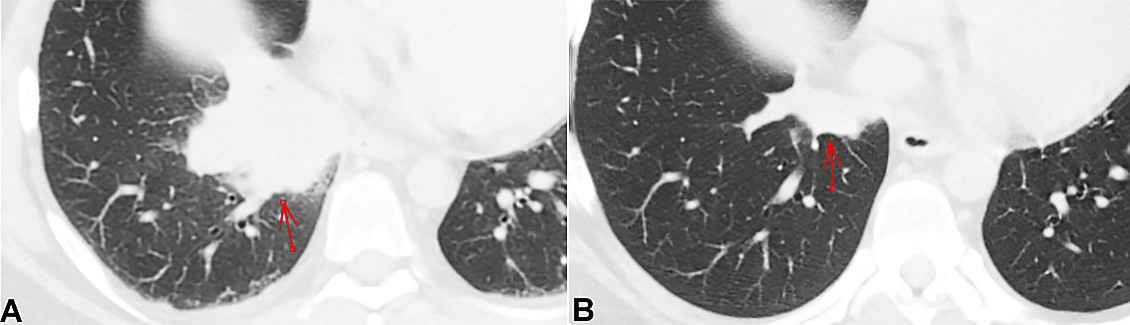
Axial lung window non-enhanced CT scan images of the chest show a significant decrease in the size of the right lower lobar lobulated metastatic mass post stereotactic radiation therapy and a dose of Atezolizumab (**B**) when compared to the images done prior (**A**).

**Figure 3:**
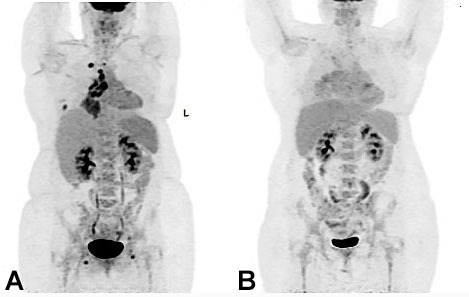
MIP PET images performed prior to the administration of Atezolizumab (**A**) and around 4 months later (**B**) demonstrate resolution of the FDG avid mediastinal and hilar lymph nodes, with almost complete resolution of the right apical and lower lobar nodules. L: Left.

The patient was diagnosed with an immune-mediated meningoencephalitis in the absence of confirmed infectious etiology. She was started on high dose steroids with dexamethasone 24 mg daily (8 mg every 8 hours), which led to a dramatic and rapid improvement. She got extubated, progressively regained her normal level of consciousness in just a few days, and was discharged 2 weeks after admission with only mild lower extremity weakness and numbness. She continued her radiotherapy, but Atezolizumab was permanently discontinued. PET CT scan done 1 month after radiotherapy showed resolution of the large right infrahilar lobulated mass, which was 5.6 cm with SUV of 9.8 and a decrease in the SUV of the large cluster of avid right paratracheal lymph nodes from 15.5 to 6.8. All small bilateral cervical nodes and bilateral inguinal nodes had fully resolved (Figures 2 and 3).

As per the patient, the only thing that she recalled was that she presented to the emergency department for fever. She then woke up in the ICU without any awareness about what had happened. Recovery was tough, but she made it through with the help of her doctors, physical therapists, and her family. And even though she still had some lower extremity weakness and required a cane to ambulate, she was very thankful that she made it through.

Her disease remained stable for around a year after that. Later, she progressed and developed metastatic brain disease, treated with radiotherapy, then eventually also progressed systemically. She received two other line of chemotherapy, but eventually, the decision was to stop treatment and focus on palliative care. Unfortunately, she passed away after 5 years after the initial diagnosis due to an infection.

## DISCUSSION

Immune checkpoints are a part of an inhibitory pathway of the immune system that controls the duration and amplitude of the immune response against a certain antigen. A major mechanism of resistance by tumors is through immune-checkpoint pathways, particularly against tumor specific T cells.^3^

Atezolizumab is a monoclonal antibody that targets PDL-1, which is expressed by tumor cells to avoid destruction by the immune system. It has already been used in urothelial, small cell, and non-small cell carcinomas but just recently got its approval for metastatic triple-negative breast cancer in 2019.^2^

All ICI can cause irAE as they produce an inflated inflammatory response. Some of the most common events are skin-related (30%–55% of patients), like rash, pruritis or dermatitis, and gastrointestinal (12%–37% of patients) including diarrhea and colitis. However, neurological adverse events are rare with an incidence ranging from 3.8 to 6.1% with ICI monotherapy, and up to 12% with combination therapy, but the incidence of Grades 3 or higher events is <1%.^4^ ^,^ ^5^

Only a few cases of meningoencephalitis due to Atezolizumab have been reported in the literature (Table 1).

**Table 1:** Similar Case reports of meningoencephalitis due to Atezolizumab in the literature, along with patient characteristics, findings, and outcomes

	Malignancy	Age (y)	Onset of symptoms	Therapy	MRI	CSF	Outcome
1^6^	Cervical	53	13 days after 1^st^ dose	Steroids	diffuse leptomeningeal enhancement	leukocytes: 553 mcL, neutrophils: 91%,	Discharged to Hospice
2^7^	Lung	78	13 days after 1^st^ dose	Steroids	No abnormal findings	high cell count of 139/μL	Improved
3^8^	Bladder	59	12 days after 1^st^ dose	Steroids	Unremarkable, Repeat MRI showed a 1-cm CNS metastasis	Rare lymphocytes	Recovery with residual weakness, then died of progressive disease
4^9^	Bladder	49	13 days after 1^st^ dose	Steroids + IVIG	diffuse leptomeningeal enhancement	50 white blood cells	Encephalitis resolved, died from disease progression and septic shock

CSF= cerebrospinal fluid; CNS= central nervous system; MRI= magnetic resonance imaging; y= years.

To our knowledge, only 7 cases have been reported at the time of this article, and they include two patients with bladder cancer, one with lung cancer, and another with cervical cancer.^6^ ^-^ ^9^ Three cases were also mentioned in the clinical trial of Atezolizumab in triple-negative breast cancer but have not been fully published, which makes our case the fourth breast cancer case.^2^

In all the published cases, the onset of neurological symptoms occurred 13 to 14 days after the 1^st^ dose of Atezolizumab, as is the case in our patient, and they were all treated with steroids,^6^ ^-^ ^8^ except for one patient who did not respond to steroids alone, but responded to intravenous immunoglobulin (IVIG).^9^

All patients initially received empiric antibiotics and antivirals, as in our case. This is important especially at the time when the etiology of encephalitis is still not determined. In our case, we considered it to be due to Atezolizumab since all cultures and viral PCR were all negative. Leptomeningeal carcinomatosis was also ruled out by cytology, which was negative for malignant cells in the CSF. The paraneoplastic syndrome was unlikely as the neurologic symptoms occurred directly after she received the Atezolizumab, the number of lymphocytes was high in the CSF, and since all symptoms resolved quickly with the administration of steroids. Therefore, she was considered to have immune-mediated meningoencephalitis. The Atezolizumab was discontinued permanently, as was the case in other published reports (grade 4 toxicity).

### Neurological adverse events from immunotherapy

It is currently well-known that checkpoint inhibitors have a different toxicity profile than chemotherapy or targeted therapy. The incidence of irAE is also different between the different types of immunotherapy. CTLA-4 inhibitors have been shown to have higher rates and severity of irAEs than PD-1/L1 inhibitors. Neurological irAE is quite uncommon and has been described more with the CTLA-4 inhibitors or with combination immunotherapy rather than with single-agent PD1-L1 inhibitors.^4^ ^,^ ^10^ The incidence of all neurological irAE with ipilimumab and nivolumab combination is around 14% and around 1 -3% with monotherapy.^11^

The autoimmune neurological events can affect the muscles, neuromuscular junction, nerves, routes, spinal cord, and brain, at variable severities, as described below. They mostly occur within the first 4 months after ICI administration but may arise at any time.^10^

Some of the mild (grade 1-2) neurological irAE that can occur include headaches, paresthesia, or small-fiber sensory neuropathies. They can occur in 6-12% of the patients, and there is no need to discontinue the ICI in these cases.

Grade 3 and 4 neurological irAEs include the following:

1. Myasthenia gravis

Myasthenia gravis (MG) is one of the most common neurological irAEs. It can occur either as de novo presentations (72%) or as an exacerbation of pre-existing myasthenia (18,2%). The average onset was 5 to 6 weeks post-treatment initiation, and around 60% of the cases were acetylcholine receptor autoantibodies (AChRAb) positive, while the remaining were negative.^12^ Interestingly, Anti-PD1, Nivolumab, and Pembrolizumab were the most often involved in immunotherapy. Common presentations do not differ from the idiopathic forms of MG and include ptosis, diplopia, and show fatigability on repetitive nerve stimulation testing.^13^

The prognosis of MG induced by ICI can be poor. In one review article, 14 out of 33 patients died after the diagnosis of MG. However, the prognosis did not seem to be related to the preexistence of myasthenia or to the presence of anti-acetylcholine receptor antibodies.^14^ Many patients required more than 1 treatment, they all received corticosteroids and prostigmine, but some of them required plasmapheresis and immunoglobulins. Rarely, azathioprine and mycophenolate mofetil were used.^14^

According to the National Comprehensive Cancer Network (NCCN) guidelines, management of grade 2 toxicity includes first to hold the immunotherapy, then to start pyridostigmine and oral prednisone. Once symptoms improve, these agents can be tapered, and immunotherapy might be resumed. Grade 3-4 symptoms will require hospitalization, immediate discontinuation of the immunotherapy (most often permanent), and higher doses of steroids. For patients who are refractory, plasmapheresis, or IVIG should be considered. All medications that can worsen MG should be avoided.^15^

1. Peripheral neuropathies

Immune-mediated polyneuropathies can occur either in an acute or chronic form. They have been mostly described as complications of ipilimumab but also of nivolumab or pembrolizumab to a lesser extent. Both sensory and motor peripheral neuropathies have been reported and are quite rare, occurring in less than 1% of patients. In grade 1 neuropathy, which is most cases, the immune checkpoint inhibitor can be continued with careful follow-up. However, if symptoms worsen and start interfering with daily activities, then it is advised to discontinue treatment and start steroids. GABA analogs can also be used for neuropathic pain control.^15^ ^,^ ^16^

1. Guillain Barre syndrome (GBS)

GBS induced by ICI is quite rare, with only a few cases reported in the literature. The agents implicated mostly were Ipilimumab and Nivolumab either alone or in combination. The presentation is typical for GBS, with sensory loss, quadriparesis, and areflexia, and the diagnosis was confirmed by lumbar puncture findings and Electromyography (EMG).^17^ ^,^ ^18^ In one case series, patients were treated with IVIG, corticosteroids, tacrolimus, or mycophenolate either alone or in combination. GBS was fatal in a few patients as 3 out of 13 patients died of respiratory insufficiency and multiorgan failure.^17^ ^,^ ^18^ Idiopathic GBS treatment does not include corticosteroids; however, cases of GBS arising as an irAE have been reported to respond differently to steroid therapy than idiopathic GBS.^18^

Similarly, rare cases of Chronic inflammatory demyelinating polyradiculoneuropathy (CIDP) as an irAE have been described. Again, the agent responsible was mainly a combination Ipilimumab and Nivolumab, and unlike GBS, some patients initially presented with headaches and neuropathic pain in the extremities, and then were diagnosed with CIDP based on the evolution of the clinical picture, EMG, MRI, and LP findings. They were treated similarly to GBS with IVIG and corticosteroids, and outcomes were favorable.^19^

Current NCCN guidelines recommend treatment of immune-related GBS by either plasmapheresis or IVIG along with pulse methylprednisolone of 1 g daily for 5 days. Unlike MG, immunotherapy should be stopped permanently and cannot be resumed once the symptoms resolve.^15^

1. Aseptic Meningitis

Aseptic meningitis occurred in around 0.1 – 0.2% of patients, and typically 1 to 7 weeks after the first dose of ICIs. Patients usually present with a stiff neck, fever, and headaches, but without any altered mental status. For diagnosis, it is essential to rule out all infectious causes. CSF findings typically show lymphocytic meningitis along with possibly meningeal enhancement on MRI. All reported cases of aseptic meningitis to date have recovered completely with steroids, and the agents responsible were mainly Ipilimumab and Nivolumab.^10^ ^,^ ^20^ ^,^ ^21^

Management of aseptic meningitis as per NCCN recommends holding the immunotherapy if mild to moderate, however, to permanently discontinue if severe. Treatment should include IV acyclovir until the viral infection is ruled out by PCR, and corticosteroids.^15^

1. Encephalitis

Among ICI-related irAEs, encephalitis is rare, but it may be severe and potentially fatal. It occurs in 0.1 – 0.2% of patients, usually within days or few weeks after ICPI initiation. Most published cases were cases of melanoma in whom the encephalitis have occurred with the combination of ipilimumab and nivolumab.^10^

The presentation was typical of encephalitis but quite heterogenous as well; it included fever, headaches, altered mental status, confusion, gait instability, and seizures. The time of onset varied; in most of the cases, it occurred just a few days after the administration of the ICI, while in one case, for example, it was 297 days after its administration. The diagnosis was made by LP, which showed lymphocytosis, MRI findings which showed hyperintensity on T2 or were unremarkable, and by ruling out infectious etiologies by cultures and PCR. Most of the cases were treated empirically with antivirals and broad-spectrum antibiotics until an infectious process was ruled out and ultimately managed with intravenous steroids. Only a few patients required IVIG. The outcome was favorable in most cases, and symptoms resolved in a few days, but there are some rare cases reported where the encephalitis was fatal.^5^ ^,^ ^16^ ^,^ ^20^

As mentioned above in our case discussion, there were also 7 cases reported of encephalitis with the use of Atezolizumab. The outcome was favorable in all of them.

Based on these reports, current guidelines recommend stopping the immunotherapy and only consider rechallenge if the symptoms were mild and completely resolved. The treatment includes steroids, and IVIG can be considered if the symptoms do not resolve on corticosteroids alone. Rituximab can also be considered in severe cases or if no or limited improvement on the mentioned therapies.^15^

### Predictor of disease response

Although our patient had a severe immune adverse reaction to the Atezolizumab, her continued remission after the meningoencephalitis could at least be partially related to a durable response to Atezolizumab. This raises the question of whether a relationship exists between the incidence and severity of the adverse events and the response to treatment.

There is some evidence that suggests that developing neurological irAE may be a positive predictor of disease response.^13^ ^,^ ^21^ ^,^ ^22^ In one cohort, the objective response rate was 70% in patients who developed neurological irAE, and the median overall survival was 45.7 months versus 11.2 months in those without neurological irAEs.^13^ ^,^ ^21^ In another case series, objective responses occurred in 50% of the patients (6/12) with 25% complete responses, despite the fact that these patients received high dose steroids to manage the toxicities.^22^

However, these are anecdotes and uncontrolled data, most of which occurred in patients with melanoma, so an accurate conclusion cannot be drawn, but the possibility that the development of neurological irAE may be associated with a better ORR remains.

## CONCLUSION

As a conclusion, even though neurological irAEs are rare, it is important to recognize and manage them appropriately as they could be fatal. Encephalitis, in most cases, can be managed with steroids alone with an excellent prognosis. The question remains whether the occurrence of neurological irAE and its severity can be related to a better tumor response or not.
